# The Influence of Ultrasound-Guided Blocks for Shoulder and Knee Surgeries on Continued Opioid Use: A 6-Month Clinical Review

**DOI:** 10.3390/jcm14144827

**Published:** 2025-07-08

**Authors:** Caroline E. Gibbs, Shahab Ahmadzadeh, Shivam S. Shah, Claudia E. Rodriguez, Anushka Singh, Hunter M. Schwab, Gabrielle A. Cassagne, Kimberly L. Skidmore, Sahar Shekoohi, Alan D. Kaye

**Affiliations:** 1 School of Medicine, Louisiana State University Health Sciences at Shreveport, Shreveport, LA 71103, USA; cgi001@lsuhs.edu (C.E.G.); sss002@lsuhs.edu (S.S.S.);; 2 Department of Anesthesiology, Louisiana State University Health at Shreveport, Shreveport, LA 71103, USA

**Keywords:** opioid use, ultrasound-guided blocks, shoulder and knee surgeries

## Abstract

**Background**: The opioid epidemic has highlighted the need for alternative pain management modalities in postoperative patients. Peripheral nerve blocks (PNBs) have been shown to reduce opioid consumption in the immediate postoperative period, but limited data exists on their impact on chronic opioid use. **Objective**: The present investigation focused on the use of preoperative PNB utilization in orthopedic surgeries and its association with chronic opioid use. **Methods**: A retrospective cohort study was conducted on 533 patients that had a total shoulder arthroplasty, reverse total shoulder arthroplasty, or knee arthroscopy between July 2021 and July 2024. Patients were grouped based on whether they received a preoperative PNB. Opioid prescription data were collected at 1-, 3-, and 6-month postoperative periods. In addition, a subset of patients completed a questionnaire to assess self-reported opioid consumption and other analgesic usage. **Results**: Patients who received a PNB were significantly less likely to report continued opioid use at one month postoperatively compared to those who did not (32.8% vs. 61.9%). Additionally, PNB recipients more often declined additional opioids due to a lack of need (*p* = 0.025), while those without a PNB cited other reasons, including fear of addiction or poor pain control (*p* = 0.033). **Conclusions**: The results of the present investigation suggest that preoperative PNBs may be associated with reduced chronic opioid use and have an important role in prescribing practices and pain management strategies following orthopedic surgery. **Limitations**: The limitations are as follows: retrospective design; potential recall and selection bias from questionnaire use; lack of data confirming actual opioid prescription fills; inclusion of patients with chronic pain comorbidities requiring long-term opioid use.

## 1. Introduction

The opioid epidemic is a public health crisis that has led to substantial morbidity and mortality in the United States. According to the most recent data from the National Institute on Drug Abuse, opioid-related deaths, including prescription opioids, rose from 49,860 in 2019 to 81,806 in 2022 [1]. Postsurgical patients are at high risk for chronic opioid use, and postoperative opioid prescriptions may be a notable factor in the ongoing crisis. One study found that within 90 days of both minor and major surgical procedures, 6% of patients refilled an opioid prescription [2]. In this regard, the opioid crisis has pushed both legislature and physicians to investigate alternative methods to control acute pain after surgery and help mitigate the risks of opioid use. The current literature has shown that using regional anesthesia can decrease opioid use in the immediate 24–72 h postoperatively, but fewer studies investigate how opioid consumption is affected thereafter [3,4]. A 2024 systematic review examining the influence of perioperative regional anesthesia on persistent opioid use and chronic pain in patients undergoing noncardiac surgery found a significant reduction in postsurgical pain for up to six months in patients who received regional anesthesia. Although this suggests a potential association with decreased opioid use, given the established link between chronic pain and opioid consumption, it did not provide direct evidence of reduced opioid utilization [5]. Another study evaluating peripheral nerve blocks (PNBs) in elective shoulder surgery found no significant association between PNB use and decreased opioid prescription fulfillment at 90 days postoperatively. These findings demonstrate the uncertainty of how regional anesthesia, specifically PNBs, affects postoperative opioid consumption beyond the immediate postsurgical recovery period [6].

Ultrasound-guided PNBs were introduced as early as 1978 and have increased in popularity over the past four decades [7]. The incorporation of PNBs into ERAS (Enhanced Recovery After Surgery) protocols is due to their demonstrated benefits over the use of systemic analgesics alone in the postoperative period [8]. These advantages include reduced opioid consumption, improved pain scores, and shorter hospital stays [7]. While shown to be effective and generally safe, potential complications include infection, hematomas, nerve injury, and local anesthetic systemic toxicity (LAST) [9,10]. Interscalene blocks are commonly used prior to upper limb and shoulder surgeries. Common complaints include paresthesia, pain, and dysesthesias, and occasionally more serious complications like phrenic nerve palsy [5,11]. Persistent phrenic nerve palsy is an uncommon complication, with one study estimating its incidence at 0.06% [12]. Special care should be taken in performing interscalene blocks in patients with underlying pulmonary comorbidities due to this potential complication. Many different PNBs are utilized for lower limb surgery with adductor canal blocks commonly chosen for knee arthroscopies. Potential complications of adductor canal blocks include failed nerve block, nerve injury, and infection [13].

The lack of definitive evidence regarding the influence of PNBs on chronic opioid consumption motivates the primary objective of this study, which is to examine differences in postoperative opioid consumption related to preoperative PNB utilization during total shoulder arthroplasty (TSA), reverse total shoulder arthroplasty (RTSA), and knee arthroscopy surgeries. We hypothesize that controlling acute pain after orthopedic surgeries could be associated with a reduction in the rate of chronic opioid use.

## 2. Subjects and Methods

**Figure 1:**
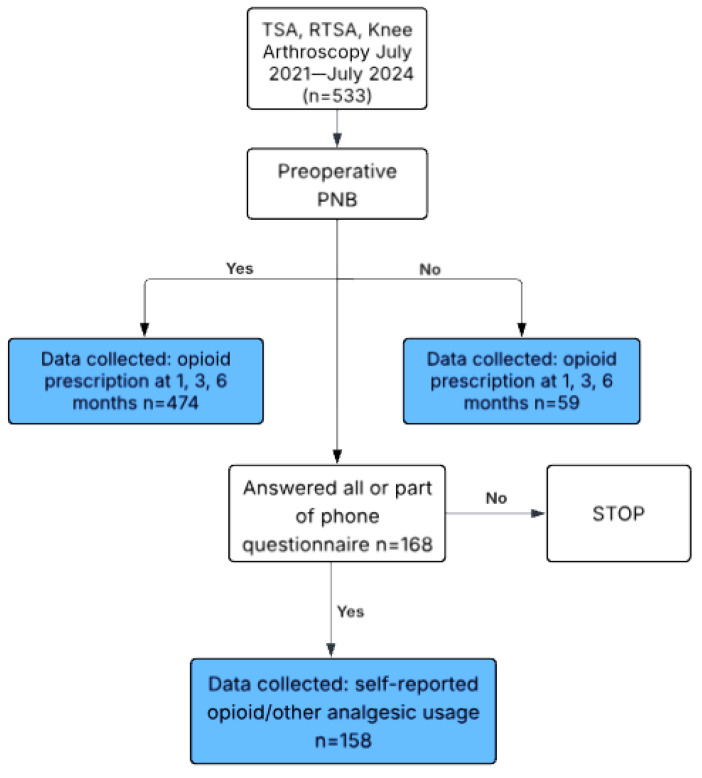
Enrollment.

The present investigation involved a retrospective study to evaluate postoperative opioid use among patients who underwent a TSA, RTSA, or knee arthroscopy over a three-year period from 1 July 2021 to 1 July 2024. Patient data were obtained from the electronic medical record system (EPIC). Individuals were stratified based on receipt of a preoperative PNB and whether they were prescribed an opioid refill within 1, 3, and 6 months following surgery. In addition, a questionnaire was administered to patients who provided informed consent. The questionnaire collected self-reported information regarding opioid consumption and use of other analgesics at the 1-, 3-, and 6-month postoperative time periods. The analgesics that were assessed included acetaminophen, ibuprofen, naproxen, celecoxib, indomethacin, aspirin, kratom, and cannabis. Furthermore, the questionnaire collected data on the perceived effectiveness of each pain management modality (Figure 1). 

The study was overseen by the Louisiana State University Health Shreveport Institutional Review Board (STUDY00002637). Patients meeting inclusion criteria were enrolled under a HIPAA waiver of informed consent. Excluded populations included neonates, pregnant individuals, and prisoners. No vulnerable populations were specifically targeted in this study. Patients included in the questionnaire were informed of the study’s purpose and voluntarily provided data. Data analysis was performed to determine statistical significance using SPS (version 29.0.2.0) Chi-Square (χ^2^), Fisher’s exact tests, and independent samples *t*-tests were used to assess the primary outcomes.

## 3. Results

A total of 533 patients underwent a TSA, RTSA, or knee arthroscopy procedure and were subsequently enrolled in the study. Among these, 269 patients underwent TSA, 126 underwent an RTSA, and 138 underwent a knee arthroscopy (Table 1). Of the cohort, 474 patients received a preoperative PNB, with details of block types highlighted in Table 2. Most patients undergoing a TSA or RTSA received an interscalene block, while most knee arthroscopy patients received an adductor canal block plus or minus IPAC/popliteal sciatic block.

Patient demographics and chronic comorbidities are shown in Table 3. The mean (SD) age at procedure date was 55.3 (0.7) years, and 46.9% were male. Average (SD) body mass index (BMI) was 31.0 (8.3), with 263 (49.3%) patients classified as having obesity (BMI > 30). The most common chronic comorbidity was hypertension, present in 321 patients (60.2%), while 97 patients had type 2 diabetes (18.2%), and 41 patients (7.7%) had chronic obstructive pulmonary disease (COPD). There were no significant differences between the patients with and without nerve blocks in most of the demographic categories and comorbidities (Table 4). However, there was a significant difference in the presence of COPD, with patients without a nerve block having a stronger association with COPD compared to those with a nerve block (*p* < 0.001). 

Postoperatively, 75 (15.8%) patients who received a preoperative PNB were prescribed an opioid refill within 1 month, 16 (3.4%) within 3 months, and 6 (1.3%) within 6 months (Table 5). Of the 59 (11.1%) patients that did not receive a preoperative PNB, 6 (8.5%) were prescribed opioids within 1 month, 3 (5.1%) within 3 months, and 1 (1.7%) within 6 months. 

**Figure 2:**
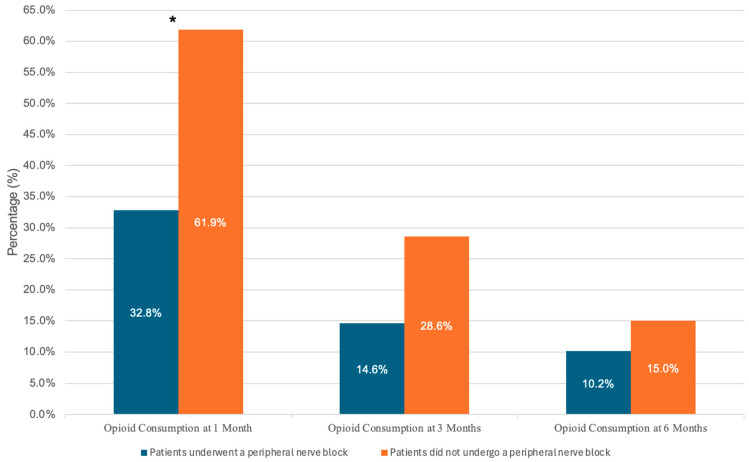
Bar graph of self-reported opioid consumption at 1 month, 3 months, and 6 months. The blue bars depict the percentage of patients with a peripheral nerve block that were still consuming opioids at the respective time period, and the orange bars depict the percentage of patients that did not undergo a peripheral nerve block that were still consuming opioids at the respective time period. The number at the center of each bar represents the percentage of individuals that were still consuming opioids during the time period. The star above the bars at 1 month indicates a *p* value less than 0.05, indicating a significant difference between the groups.

**Figure 3:**
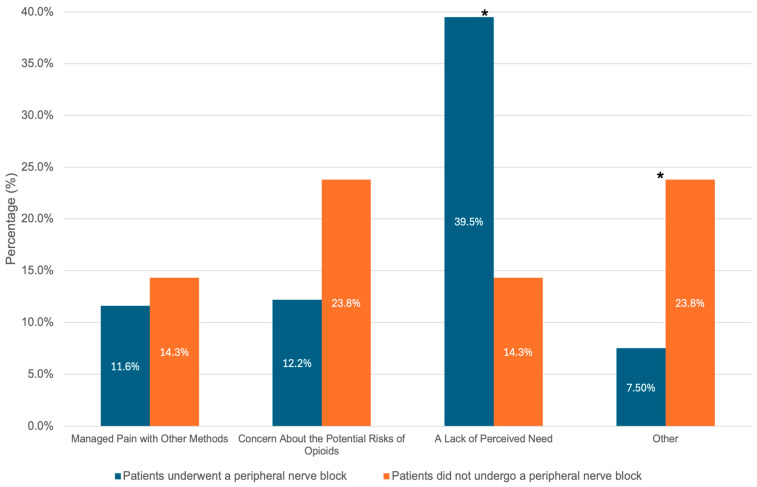
Bar graph of self-reported reasons for declining future opioid prescriptions. Patients were given a series of responses to choose from, which included managing pain with other methods, concern about the potential risks of opioids, a lack of perceived need, or other reasons. The blue bars depict the percentage of patients with a peripheral nerve block that reported the corresponding response, and the orange bars depict the percentage of patients that did not undergo a peripheral nerve block that reported the corresponding response. The number at the center of each bar represents the percentage of individuals that reported the corresponding response as the reason for declining future opioid prescriptions. The stars above the bars indicate a *p* value less than 0.05, indicating a significant difference between the groups.

A further analysis of the questionnaire responses provided additional insight into postoperative opioid and analgesic consumption. Of the total 533 patients enrolled, 168 patients answered all or partial components of the questionnaire. A total of 158 patients provided data for their postoperative opioid consumption at the 1- and 3-month time intervals, and 157 patients provided this data at the 6-month time interval (Figure 2 and Table 6). Of the 158 patients, 58 (36.7%) reported still taking opioids at 1 month and 26 (16.5%) at 3 months, while only 17 (3.8%) of the 157 patients reported still taking opioids at 6 months. Patients who did not receive a PNB were significantly more likely to report continued opioid usage at one month compared to those who received a PNB (62% vs. 33%, *p* = 0.01). Additionally, a similar percentage of patients reported a desire to decline further opioid prescriptions in patients who received a PNB compared to those without a PNB (72.2% vs. 75.0%, *p* = 0.792). However, the reason for declining future opioid prescriptions was different for each of these groups. Patients who received PNB were more likely to decline further opioid prescriptions due to a lack of perceived need (*p* = 0.025), whereas those without a PNB were more likely to respond with “other” reasons for declining additional opioids (*p* = 0.033) (Figure 3).

## 4. Discussion

The opioid epidemic continues to pose a substantial challenge to perioperative care, especially in orthopedic surgery, which is a specialty historically associated with some of the highest rates of opioid prescribing [14]. In this retrospective study, we assessed whether preoperative PNBs influence long-term opioid consumption and patient perception in 533 patients undergoing TSA, RTSA, or knee arthroscopy. The evaluation of PNB efficacy on reducing opioid usage in the long-term postoperative period is of interest for future studies as the current literature is limited to short-term outcomes [15,16,17]. Our results suggest that PNBs may play a key role in reducing opioid consumption in the first month following surgery, while also impacting patient-reported reasons for declining further opioid use.

The most notable finding was a statistically significant reduction in self-reported opioid use at one month postoperatively in patients who received a PNB compared to those who did not (32.8% vs. 61.9%, *p* = 0.01). While most of the existing literature focuses on the short-term postoperative period, this finding suggests that the benefits of PNBs in reducing opioid reliance may extend beyond the first few days following orthopedic surgery. The 1-month outcome aligns with prior research showing that regional techniques, when incorporated into a multimodal approach, may be associated with reduced opioid requirements [18]. A prospective, multi-center study by Sethi et al. found that 80% of patients undergoing a TSA required 15 or fewer oxycodone 5 mg pills after receiving a multimodal approach, including regional nerve blocks, for pain management [18].

However, no significant differences in opioid use were observed at the 3- and 6-month time periods, which potentially limits the evidence supporting PNBs as a strategy for reducing chronic opioid use. This lack of significance could be attributed to resolution of surgical pain in both groups over time, greater reliance on non-opioid analgesics, or limited sample size. Additionally, it is important to note that PNBs are not without clinical limitations. The significant association of COPD and the absence of a nerve block aligns with previous research that found that certain nerve blocks, particularly interscalene blocks, are contraindicated in patients with COPD due to the risk of phrenic nerve palsy [19,20]. Obesity is another common reason associated with not receiving a PNB, as it can increase the depth of target nerves and present positioning challenges during block placement, although this was significantly reflected in our data [21]. Despite these considerations, strategies that limit opioid exposure early in recovery, such as PNBs, may still contribute to reduced overall opioid consumption and help lower the risk of persistent opioid use. 

Our data found that more than 20% of patients received an opioid prescription within 6 months after receiving a PNB. However, we found a discrepancy between prescription data and the self-reported opioid consumption that was found using our follow-up questionnaire, especially during the 1-month time period. While only 15.8% of PNB recipients and 8.5% of non-PNB patients were prescribed opioids at 1 month (*p* = 0.136), a significantly higher proportion reported actually taking opioids at that time (32.8% and 61.9%, respectively). Importantly, the difference in sample size between the prescription data (*n* = 533) and self-reported data (*n* = 158), due to survey nonresponse, may account for discrepancies between these datasets. No significant differences were found between PNB recipients and non-PNB recipients in self-reported opioid consumption from a family member or friend, or using leftover opioids from a previous surgery or procedure at any time point (1, 3, or 6 months postoperatively). This mismatch may also reflect a potential trend of under prescribing, in which patients experiencing moderate to severe pain do not receive continued opioid therapy, despite an ongoing need. Additionally, some patients may extend the use of an initial prescription over time, rationing their doses due to hesitancy to request refills or difficulty obtaining them. 

One contributing factor to this gap may be evolving prescribing behaviors among orthopedic surgeons. Orthopedic surgeons have historically been the top specialty in opioid dispensing, but in recent years there has been a concerted effort to decrease the volume and duration of opioid prescriptions, primarily in response to increasing public health scrutiny and a growing awareness of opioid misuse [22,23]. While this shift is definitely needed to combat the opioid crisis, our results suggest that it may have unintended consequences such as an overcorrection, leading to insufficient pain control in select patients. These findings raise the possibility that some orthopedic surgeons may be reluctant to authorize opioid refills, even when pain persists, and ongoing opioid use is medically justified. This highlights the need for balanced prescribing practices, where legitimate analgesic needs are met while also minimizing excess opioid exposure.

Additionally, differences in patient perception provide further insight into how PNBs can alter the postoperative experience. Among patients who declined further opioid prescriptions, those who received a PNB were more likely to report doing so due to a lack of perceived need (*p* = 0.025), while patients who did not receive a block more often cited “other” reasons (*p* = 0.033), such as fear of addiction or dissatisfaction with pain control. 

This distinction suggests that PNBs are associated with reduced pain intensity, greater patient confidence in non-opioid alternatives, and lower perceived pain levels, which may contribute to safer postoperative recovery.

Among patients receiving a PNB, 13.5% of those undergoing upper extremity surgery required an opioid refill at one month, compared to 20.6% following lower extremity surgery (*p* = 0.062). This trend may reflect differences in the type and efficacy of the regional anesthesia used. Adductor canal blocks, which were the most common nerve block used for knee arthroscopies, primarily target anterior knee pain and do not include a long-acting bupivacaine (Exparel), unlike interscalene blocks, which may have provided more prolonged analgesia.

These findings demonstrate the value of incorporating PNBs into multimodal pain protocols and highlight the need for better alignment between pain control needs and prescribing behaviors. Clinicians should feel comfortable assessing pain on an individual basis instead of defaulting to minimal or no refills due to broader systemic concerns. Although these concerns are important, providers should try to find a safe balance between ensuring that a patient’s pain needs are being met and avoiding the potential risk of opioid dependence or misuse. At the same time, patient education about expectations, safe tapering, and alternatives to opioids should continue to be a key part of safe opioid prescribing practices.

### 4.1. Future Directions

Future research should focus on prospective, randomized controlled trials to confirm the long-term benefits of PNBs in reducing opioid use and improving pain perception across various orthopedic procedures. These studies should incorporate standardized pain assessments, evaluate patient-reported outcomes, and stratify findings by procedure type, baseline opioid use, and comorbidities. Additionally, developing evidence-based prescribing guidelines, especially for opioid refills, can help balance the need for effective pain control with the goal of minimizing excess opioid exposure. The integration of PNBs with other multimodal strategies, including physical therapy, NSAIDs, acetaminophen, and behavioral interventions, should also be further studied to determine optimal combinations for postoperative recovery. Finally, provider-focused interventions, such as continuing education and an emphasis on personalized pain management, may improve prescribing practices and help close the observed gap between actual opioid consumption and physician prescribing. These strategies offer promising opportunities to create safer, more personalized, and effective postoperative pain management strategies.

### 4.2. Limitations

There are several limitations to this study. Importantly, a significant portion of the opioid consumption data was obtained through patient self-reported questionnaires, which introduces the potential for recall bias. Patients may have misremembered, incorrectly recalled, or overestimated actual opioid use at the 1-, 3-, and 6-month time points, especially if they were attempting to ration medications or relied on multiple analgesic methods. Additionally, only a subset of the total cohort completed the survey, which may reflect response bias, because those with stronger opinions or more memorable experiences may have been more likely to respond. The retrospective design further limits the ability to establish causality between PNB use and reduced opioid consumption. Although prescription refill data was available from the electronic medical record, this data does not show whether the prescribed opioids were consumed, and it does not reflect any medications obtained outside the system. Also, a small number of patients in the cohort had chronic pain conditions or were already on long-term opioid therapy prior to surgery. Although this only affected a few patients, this may have caused the results to be skewed. Additionally, opioid usage patterns may have been influenced in unmeasured ways, such as baseline pain tolerance or past opioid usage. Lastly, the limited sample size did not allow for stratified analysis by nerve block type. Future prospective studies with larger, more evenly distributed samples and the real-time monitoring of medication use are needed to address these limitations.

## 5. Conclusions

Overall, the results of the present investigation highlighted the potential benefits of preoperative PNBs in reducing postoperative opioid consumption following common orthopedic procedures. Patients who received PNB were significantly less likely to report continued opioid use at one month postoperatively compared to those who did not, indicating a potential association between PNB use and reduced risk of prolonged opioid use. Additionally, patients receiving PNBs were more likely to decline further opioid prescriptions due to a lack of perceived need, indicating improved pain control. These findings support the integration of PNBs into ERAS protocols and emphasize the importance of personalized opioid prescribing practices. However, discrepancies between prescription data and self-reported use highlight the need for closer alignment between actual pain experiences and prescribing behavior. Future prospective studies with standardized assessments and broader patient representation are needed to confirm these findings and guide evidence-based postoperative pain management.

## Figures and Tables

**Table 1:** Surgical details (*n* = 533).

Total shoulder arthroplasty, No. (%)	269 (50.5)
Reverse total shoulder arthroplasty, No. (%)	126 (23.6)
Knee arthroscopy, No. (%)	138 (25.9)
PNB given, No. (%)	474 (88.9)

**Table 2:** Peripheral nerve block (PNB) characteristics (*n* = 474).

Block Type	No. (%)
Adductor canal	78 (16.5)
Adductor canal and popliteal	40 (8.4)
Interscalene	341 (71.9)
Suprascapular	7 (1.5)
Other	8 (1.7)

Note: Other blocks include adductor and IPACK (*n* = 4), popliteal (*n* = 1), femoral (*n* = 1), sciatic, adductor, and popliteal (*n* = 1), and adductor canal, IPACK, and femoral (*n* = 1).

**Table 3:** Patient demographics and chronic comorbidities (*n* = 533).

Age at procedure date, mean (SD), y	55.3 (0.7)
Male, No. (%)	250 (46.9)
Body mass index, mean (SD)	31.0 (8.3)
Obesity, No. (%)	263 (49.3)
Hypertension, No (%)	321 (60.2)
Type 2 diabetes, No. (%)	97 (18.2)
Chronic obstructive pulmonary disease, No. (%)	41 (7.7)

**Table 4:** Differences in patient demographics and chronic comorbidities between groups (*n* = 533).

	Nerve Block	No Nerve Block	*p* Value *
Age in years at procedure date, mean (SD)	54.88 (17.437)	57.90 (17.533)	0.211
Male, No. (%)	229 (48.3)	21 (35.6)	0.065
Body mass index, mean (SD) (*n* = 515)	30.96 (8.26)	31.79 (8.99)	0.485
Obesity, No. (%)	232 (48.9)	31 (52.5)	0.602
Hypertension, No (%)	282 (59.5)	39 (66.1)	0.328
Type 2 diabetes, No. (%)	83 (17.5)	14 (23.7)	0.243
Chronic obstructive pulmonary disease, No. (%)	29 (6.1)	12 (20.3)	**<0.001**

* *p* values calculated using χ^2^ test or independent samples *t*-test.

**Table 5:** Opioid prescription outcomes at 1, 3, and 6 Months Postoperatively (*n* = 533).

Outcome	Total, No. (%)	Nerve Block (*n* = 474), No. (%)	No Nerve Block (*n* = 59), No. (%)	*p* Value *
Opioid prescription at 1 mo	80	75 (15.8)	5 (8.5)	0.136
Opioid prescription at 3 mo	19	16 (3.4)	3 (5.1)	0.456
Opioid prescription at 6 mo	7	6 (1.3)	1 (1.7)	0.562

Abbreviation: mo, month. * *p* values calculated using χ^2^ test or Fisher’s exact test.

**Table 6:** Opioid usage outcomes at 1, 3, and 6 months postoperatively.

Outcome	Total, No. (%)	Nerve Block, No. (%)	No Nerve Block, No. (%)	*p* Value *
Opioid consumption at 1 mo (*n* = 158)	58 (36.7)	45 (32.8)	13 (61.9)	**0.010**
Opioid prescription at 3 mo (*n* = 158)	26 (16.5)	20 (14.6)	6 (28.6)	0.119
Opioid prescription at 6 mo (*n* = 157)	17 (3.8)	14 (10.2)	3 (15.0)	0.457

Abbreviation: mo, month. * *p* values calculated using χ^2^ test or Fisher’s exact test.

## Data Availability

All the data are included in the manuscript.
